# Comprehensive Analysis of miRNA and mRNA Expression Profiles during Muscle Development of the Longissimus Dorsi Muscle in Gannan Yaks and Jeryaks

**DOI:** 10.3390/genes14122220

**Published:** 2023-12-15

**Authors:** Dashan Guo, Yali Wei, Xupeng Li, Yanbin Bai, Zhanxin Liu, Jingsheng Li, Zongchang Chen, Bingang Shi, Xiaolan Zhang, Zhidong Zhao, Jiang Hu, Xiangmin Han, Jiqing Wang, Xiu Liu, Shaobin Li, Fangfang Zhao

**Affiliations:** Gansu Key Laboratory of Herbivorous Animal Biotechnology, College of Animal Science and Technology, Gansu Agricultural University, Lanzhou 730070, China

**Keywords:** Jeryak, longissimus dorsi, small RNA-seq, miRNA, production performance

## Abstract

A hybrid offspring of Gannan yak and Jersey cattle, the Jeryak exhibits apparent hybrid advantages over the Gannan yak in terms of production performance and other factors. The small non-coding RNAs known as miRNAs post-transcriptionally exert a significant regulatory influence on gene expression. However, the regulatory mechanism of miRNA associated with muscle development in Jeryak remains elusive. To elucidate the regulatory role of miRNAs in orchestrating skeletal muscle development in Jeryak, we selected longissimus dorsi muscle tissues from Gannan yak and Jeryak for transcriptome sequencing analysis. A total of 230 (DE) miRNAs were identified in the longissimus dorsi muscle of Gannan yak and Jeryak. The functional enrichment analysis revealed a significant enrichment of target genes from differentially expressed (DE)miRNAs in signaling pathways associated with muscle growth, such as the Ras signaling pathway and the MAPK signaling pathway. The network of interactions between miRNA and mRNA suggest that some (DE)miRNAs, including miR-2478-z, miR-339-x, novel-m0036-3p, and novel-m0037-3p, played a pivotal role in facilitating muscle development. These findings help us to deepen our understanding of the hybrid dominance of Jeryaks and provide a theoretical basis for further research on the regulatory mechanisms of miRNAs associated with Jeryak muscle growth and development.

## 1. Introduction

The Jeryak is the result of crossbreeding between Gannan yak and Jersey cattle. Numerous studies have demonstrated that from Jersey cattle for crossbreeding with Gannan yak, the hybrid offspring of Jeryaks exhibit significantly increased body size and weight, robust physique, strong adaptability, and rapid growth and development [1], which provides support for herders’ living production and pastoral economy [2]. In view of this, exploring the molecular regulatory mechanisms of skeletal muscle development may provide a direction to improve the production performance of Jeryaks.

The economic value of mammals is evaluated based on their meat production and its quality, which are influenced by the growth of skeletal muscle [3]. The growth of skeletal muscle is an intricate and accurate process, including the creation of fetal muscle fibers prior to delivery and the enlargement of muscle fibers after delivery [4]. Furthermore, the regulation of numerous transcription factors is imperative for the growth of skeletal muscles [5]. Muscle-derived regulatory factors (MRFs) and muscle cell enhancer 2 (MEF2) are widely recognized as crucial elements in the growth and development of skeletal muscle, as evidenced by previous studies [6,7,8]. PAX3 and PAX7 act as regulatory factors that control the development of skeletal muscle by interacting with MRFs [9]. Muscle growth inhibitor (MSTN) has been identified as a specific negative regulatory factor involved in skeletal muscle development, which interacts with certain signaling pathways to inhibit the expression of MRFs or PAX [10,11]. Furthermore, the Wnt, MAPK, and PI3K-Akt signaling pathways play a pivotal role in myogenesis, regeneration, and differentiation across multiple species including mice, chicken, and humans [12,13,14,15,16,17]. In recent years, advancements in high-throughput sequencing technology have revealed that skeletal muscle growth is influenced not only by protein-coding RNAs but also by non-coding RNAs (ncRNAs), specifically microRNAs (miRNAs). These ncRNAs play a significant role in various biological processes and are regulated through multiple mechanisms.

MiRNAs, a class of small RNAs that are highly conserved across evolutionary species, exert post-transcriptional regulation on target genes by either degrading or inhibiting the translation of specific messenger RNAs (mRNAs) [18,19]. They have been demonstrated to play a role in vital biological processes, including cell proliferation and differentiation [20,21,22]. Currently, a plethora of research studies have indicated that the abundant expression of muscle-specific miRNAs, such as miR-1, miR-206, miR-133, and miR-499, within skeletal muscles significantly influences their development [4,23,24]. Chen et al. [25] reported an observed notable increase in the levels of miR-1 and miR-206 during satellite cells’ differentiation in skeletal muscle, while a decrease was observed during the process of skeletal muscle regeneration. Further investigation unveiled that miR-1/206 exerted inhibitory effects on the expression of *Pax7*, thereby hindering the proliferation and differentiation of satellite cells. Nakasa et al. [26] showed that the activation of *MyoD*, *MyoG*, and *Pax7* occurred when miR-1, miR-133, and miR-206 were injected together at the site of muscle injury in rats, and this activation subsequently facilitated muscle regeneration and suppressed muscle fibrosis. Meanwhile, miR-499’s significance in regulating the composition of muscle fibers has been demonstrated [27]. Additionally, miR-22-3p impedes the proliferation of skeletal muscle cells and promotes cellular differentiation by virtue of its interaction with *IGFBP3* [28]. The identified interactions between oar-mir-655-3p and oar-mir-381-5p with *ACSM3* and *ABAT* play a crucial role in the process of muscle organogenesis and myoblast migration in sheep [29]. In recent years, transcriptome sequencing has been extensively explored to investigate the correlation between miRNAs and mRNAs in skeletal muscle development. However, the regulatory mechanisms governing miRNA–mRNA interaction in relation to Jeryaks’ muscle growth and development have yet to be investigated.

Therefore, in this study, we generated six small RNA libraries by collecting samples from the longissimus dorsi muscle of Gannan yaks and Jeryaks. By employing high-throughput sequencing and conducting comprehensive bioinformatics analysis, we elucidated the expression pattern of miRNAs and finally constructed an miRNA–mRNA regulatory network related to muscle growth by co-analysis with RNA-seq. These findings help us to deepen our understanding of the hybrid dominance of Jeryaks and provide a theoretical basis for further research on the regulatory mechanisms of miRNAs associated with Jeryak muscle growth and development.

## 2. Materials and Methods

### 2.1. Sample Collection

Three male Gannan yaks (M1, M2, M3) and three male Jeryaks (P1, P2, P3) were sourced from Hezuo City, Gannan Tibetan Autonomous Prefecture, Gansu Province. They were subsequently categorized into groups M and P. All the animals were healthy, aged 4 years old, and had free access to food and water under the same feeding conditions. Prior to slaughter, the experimental animals underwent an overnight fasting period followed by adherence to established slaughterhouse protocols. The longissimus dorsi muscle was collected on the slaughtering line and samples were promptly transferred to 1.5 mL centrifuge tubes before being preserved at −80 °C for future sequencing.

### 2.2. Total RNA Extraction

We employed a TRIzol kit (Invitrogen, Carlsbad, CA, USA) for the isolation of total RNA from longissimus dorsi muscle tissue samples. Initially, we pulverized rice grain-sized tissues in a liquid nitrogen environment using a grinding apparatus. Subsequently, the crushed sample was transferred to a 1.5 mL centrifuge tube and immediately supplemented with 1000 µL of TRIzol followed by thorough mixing. After allowing it to stand at room temperature for 5 min and subsequent centrifuging at 12,000× *g* for 5 min, the supernatant was carefully aspirated and mixed with 200 µL of chloroform in a new centrifuge tube. The mixture was thoroughly mixed and left at room temperature for an additional 5 min, then centrifuged at 12,000× *g* for 15 min. Following this step, the supernatant was again aspirated and transferred to a new centrifuge tube, to which 500 µL of isopropanol was added and mixed thoroughly. The resulting mixture was left for 10 min, and then centrifuged at 12,000× *g* for 10 min before discarding the supernatant. Subsequently, an 80% ethanol was added to the centrifuge tube in a volume equivalent to that of TRIzol. The mixture was then centrifuged at 7500× *g* for 5 min and the supernatant was discarded. After drying at room temperature for a duration of 2–4 min, solubilization of RNA was achieved by adding 20 µL of RNase-free water. Finally, the sample was stored at −80 °C.

### 2.3. Construction and Sequencing of Small Ribonucleic Acid Library

After extracting the total RNA, we employed the polyacrylamide gel electrophoresis (PAGE) technique to selectively enrich small RNAs within the 18–30 nt range. Subsequently, reverse transcription and PCR amplification were performed on small RNAs ligated with both 3′ and 5′ adapters, resulting in PCR products of approximately 140–160 bp for cDNA library construction. The quality of the samples was assessed using an Agilent 2100 instrument (Agilent Technologies, Palo Alto, CA, USA) and ABI Prism 7500 real-time PCR system (Applied Biosystems, Foster City, CA, USA). Finally, sequencing was conducted on an Illumina NovaSeq 6000 instrument (Guangzhou, China).

### 2.4. Differentially Expressed miRNA Analysis

We processed the sequencing data as follows: Firstly, we filtered out reads with multiple bases of poor quality (q-value ≤ 20) or unknown nucleotides (N). Secondly, we removed the portions of reads that lacked the 3′ adapters and those containing the 5′ adapters. Subsequently, we discarded reads that contained both the 3′ and 5′ linkers without small RNA fragments in between. Additionally, any reads containing polyA tails were eliminated. Finally, we excluded reads shorter than 18 nt in length. To identify and remove non-coding RNAs such as rRNAs, scRNAs, snoRNAs, snRNAs, and tRNAs from our dataset, we compared all clean labels against small RNA databases including GenBank (v209.0) and Rfam (v11.0). Furthermore, to ensure accurate alignment with the yak reference genome (LU_Bosgru_v3.0), cleaning markers were aligned accordingly. Moreover, labels mapping to exons, introns, and repetitive sequences were also eliminated.

We employed miRDeep2 (v2.0.0.7) software [30] for the prediction of novel miRNAs while quantifying their expression levels using the TPM (TPM) algorithm. Heat maps were generated to visualize the expression profiles of miRNAs across different sample sets, and clustering was performed based on similar expression patterns. The DESeq2 (V1.20.0) [31] software was utilized to analyze the differential expression of miRNAs in Jeryak and Gannan yak populations. In the comparison, we identified miRNAs with a fold change > 2.0 and *p* < 0.05 as (DE)miRNAs.

### 2.5. Target Gene Prediction of Differentially Expressed miRNAs and KEGG and GO Analysis

We utilized both Miranda (v3.3a) [32] and TargetScan (v7.0) [33] software to perform target gene prediction for known, identified, and newly discovered miRNAs. The intersection of the prediction results was considered as the set of target genes for these miRNAs. Subsequently, we utilized the GO (http//www.geneontology.org/, accessed on 11 July 2023) and KEGG (http//www.genome.jp/kegg/, accessed on 11 July 2023) databases to determine significant biological functions and pathways associated with these targets. Significantly enriched biological processes in differentially expressed miRNA target genes were defined based on a screening threshold of *p* < 0.05.

### 2.6. miRNA–mRNA Interaction Network Construction

We performed a comprehensive analysis using RNA-seq (accession number: PRJNA1023693) to investigate the targeting relationship between miRNA and mRNA, as well as identify mRNAs associated with muscle growth. Subsequently, we assessed the correlation between miRNA expression and target genes using Pearson correlation coefficient (PCC). In cases where the expression of a target gene exhibited negative correlation with its corresponding miRNA, we considered it as a potential target gene for differentially expressed (DE)miRNAs. Finally, we employed Cytoscape 3.1.0 (http//www.cytoscape.org/, accessed on 11 July 2023) to visualize the interaction network between miRNAs and mRNAs.

### 2.7. Real-Time qPCR Validation of Differentially Expressed miRNAs

In order to confirm the accuracy of the sequencing findings, we validated qRT-PCR on the 7 differentially expressed miRNAs. We used miRNA Design V1.01 to design forward primers for miRNAs. The primers for (DE)miRNAs are listed in Table 1. Afterwards, total RNA was extracted from the samples using TrRIzol reagent (Invitrogen, Carlsbad, CA, USA). We used the miRNA First-Strand cDNA Synthesis Kit (Transgen Biology, Beijing, China) and performed reverse transcription and real-time fluorescence quantitative PCR with *U6* as the internal reference gene. First, we added total RNA, TransScript^®^ miRNA RT Enzyme Mix, 2 × TS miRNA Reaction Mix, and RNase-free water to the PCR tube to form a 20 µL reaction system. Then, this was lightly mixed and incubated at 37 °C for 1 h. The RT Enzyme Mix was inactivated by heating 20 µL of the system reaction at 85 °C for 5 s, and miRNA first-strand cDNA construction was completed. Next, we added cDNA, Forward Primer (10 µM), 2 × PerfectStart^TM^ Green qPCR SuperMix, Universal miRNA qPCR Primer (10 µM), Passive Reference Dye (50×), and RNase-free water to a new PCR tube to form a 20 µL reaction system. Real-time fluorescence quantitative PCR was completed after 40–45 cycles at 94 °C for 5 s and 60 °C for 30 s.

Next, we analyzed miRNA expression levels in different tissues to confirm that miRNAs were involved in bovine skeletal muscle development. Briefly, we isolated total RNA from heart, liver, spleen, lung, kidney, longissimus dorsi muscle, and subcutaneous fat using TRIzol (Invitrogen, Carlsbad, CA, USA). Subsequently, we performed reverse transcription and real-time fluorescence quantitative PCR using an miRNA First-Strand cDNA Synthesis Kit (Transgen Biology, Beijing, China) according to the above method. Finally, we evaluated miRNA expression using qRT-PCR with *U6* as internal reference.

All qRT-PCR reactions were performed on an ABI Prism 7500 real-time PCR system (Applied Biosystems, Foster City, CA, USA). All experiments were performed with three biological replicates and data were analyzed using the 2^−ΔΔCT^ method. Graphs were generated for all experiments using GraphPad Prism 9.

## 3. Results

### 3.1. Summary of Sequencing Small RNA

**Figure 1:**
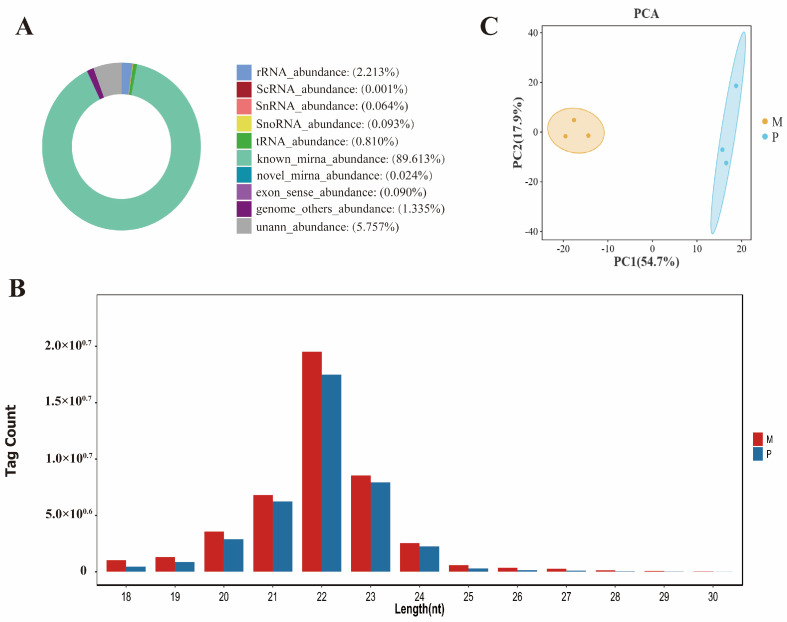
Quality control of small RNA sequencing data. (**A**) Non−coding RNA statistics. (**B**) Statistical analysis of small RNA fragment size. (**C**) Principal component analysis (PCA).

To detect (DE)miRNAs during muscle development in Gannan yaks and Jeryaks, we created and sequenced six small RNA libraries (M1, M2, M3, P1, P2, and P3) using the Illumina NovaSeq 6000 platform. A total of 17,408,914, 14,015,050, 16,597,591, 14,318,748, 9,195,586, and 16,740,804 clean readings were produced in M1, M2, M3, P1, P2, and P3, correspondingly. Following the elimination of adapters, impurities, and reads of poor quality, a total of 16,255,077, 13,362,108, 15,172,291, 13,782,038, 8,756,687, and 16,180,410 clean tags were acquired and utilized for subsequent examination (Table 2). After comparing all clean tags with GenBank and Rfam, it was discovered that a total of 74,809,937 (89.613%) known miRNAs and 19,881 (0.024%) novel miRNAs were obtained from Gannan yaks and Jeryaks once small RNAs matching rRNA, tRNA, snRNA, snoRNA, and scRNA were eliminated (Figure 1A, Table S1). Meanwhile, 72.17% and 78.05% clean tags in M and P, respectively, were mapped to the yak reference genome (LU_Bosgru_v3.0) (Table S2). The size distributions of small RNAs in the longissimus dorsi muscle of the two breeds are comparable, with the majority being concentrated around 22 nt (Figure 1B). Principal component analysis (PCA) revealed similarities between samples and differences between the two cattle breeds (Figure 1C). The presence of these findings suggests that the sequencing data are of excellent quality.

### 3.2. Differential Expression Analysis of miRNAs

**Figure 2:**
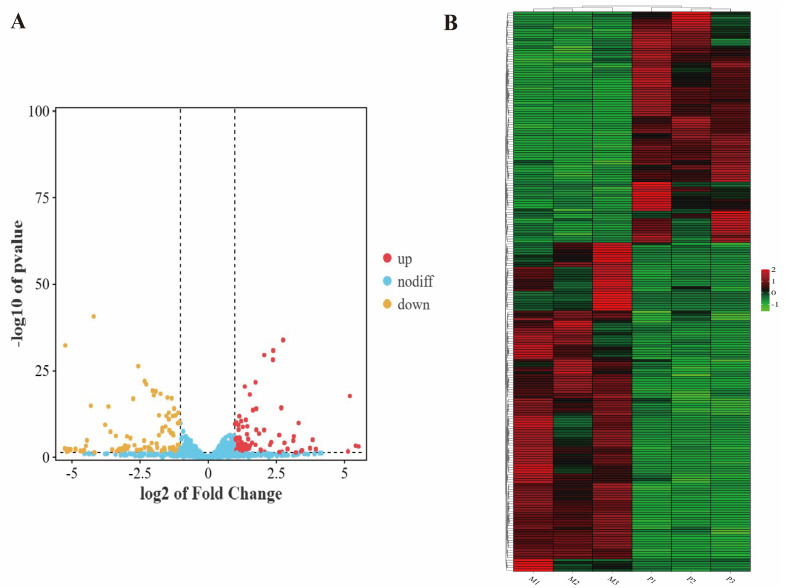
Statistical analysis of (DE)miRNAs. (**A**) The expression of miRNA volcano. The yellow dots on the left represent miRNAs that were significantly down-regulated in the P group compared to the M group; the blue dots in the middle represent miRNAs that were non−significant differences in the P group compared to the M group; and the red dots on the right represent miRNAs that were significantly up-regulated in the P group compared to the M group. (**B**) Cluster map of differentially expressed miRNAs. Red color indicates elevated expression and green color indicates decreased expression.

We compared miRNAs from groups M and P using DESeq2 (V1.20.0) software and identified 230 differentially expressed miRNAs consisting of 172 already characterized miRNAs and 58 newly discovered ones. In comparison to Gannan yaks, the longissimus dorsi muscle of Jeryaks had 135 down-regulated miRNAs and 95 up-regulated miRNAs (Figure 2A). And they were clustered in the two branches of M and P, respectively (Figure 2B).

### 3.3. Target Gene Prediction of Differentially Expressed miRNAs and KEGG and GO Analysis

**Figure 3:**
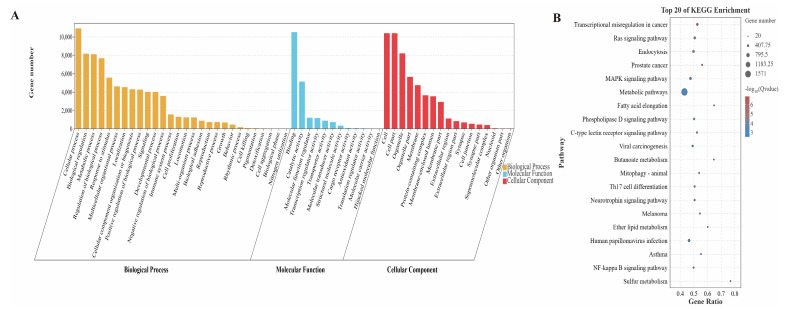
Functional enrichment analysis of (DE)miRNA target genes. (**A**) GO enrichment analysis of target genes of (DE)miRNAs. (**B**) Top 20 KEGG signaling pathways enriched by (DE)miRNAs target genes.

As is well known, miRNA controls biological processes by suppressing the translation of target genes [34]. Hence, to gain a deeper comprehension of the possible functions of miRNAs in the growth of skeletal muscle, targetscan and miRanda were employed for the anticipation of its target genes. The results showed that 17,285 target genes were predicted by the 230 (DE)miRNAs (Table S3). The analysis of GO enrichment indicated that these target genes were associated with 3217 functional categories that had been significantly enriched (*p* < 0.05) (Table S4). Among them, 270 GO terms were enriched in the cellular component classification, including cell, cell part, Wnt signalosome, PRC1 complex, striated muscle thin filament, and other terms. In the category of molecular function, there were 575 significantly enriched terms, mainly including catalytic activity, binding to growth factors, protein interaction, activity as a transcription regulator, binding to transcription factors, binding to Ras GTPase, and other related terms. There were 2372 GO terms that were significantly enriched in biological processes, primarily involving the skeletal muscle cell differentiation, regulation of smooth muscle cell migration, positive regulation of skeletal muscle tissue development, smooth muscle cell proliferation, metabolic process, and other related terms (Figure 3A). The analysis of KEGG indicated that the target genes of (DE)miRNAs showed a notable enrichment in 118 signaling pathways (*p* < 0.05) (Table S5). The enriched pathways mainly involved Ras signaling pathway, MAPK signaling pathway, Wnt signaling pathway, FoxO signaling pathway, and fatty acid metabolism(Figure 3B).

### 3.4. Building the Network for miRNA–mRNA Interactions

**Figure 4:**
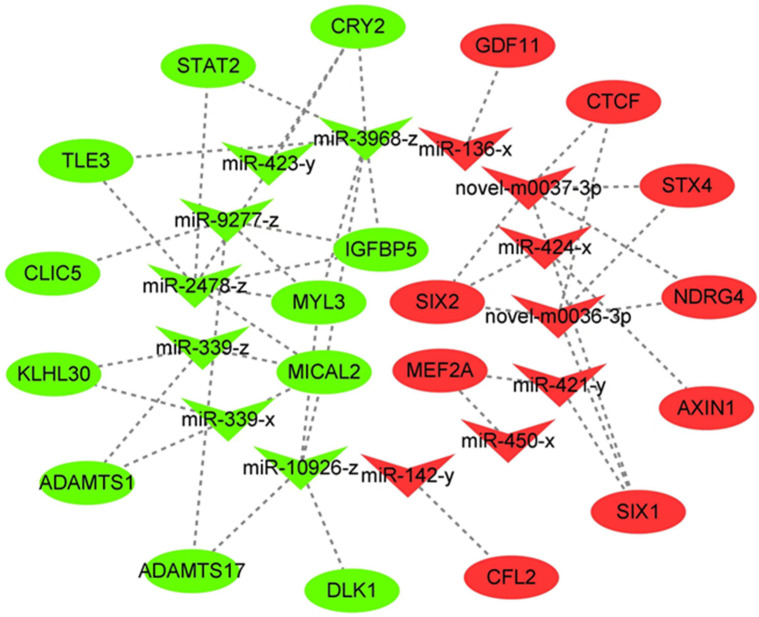
Differentially expressed miRNA–mRNA interaction network between Gannan yaks and Jeryaks. Red color represents up-regulated miRNAs and down-regulated mRNAs in Jeryak compared to Gannan yak; green color represents down-regulated miRNAs and up-regulated mRNAs in Jeryak compared to Gannan yak.

These 230 (DE)miRNAs and RNA-seq screened DEMs were analyzed jointly based on the negative regulation of mRNAs by miRNAs. To further explore the potential role of miRNAs in Jeryak muscle growth, 5916 pairs of miRNA–mRNA relationships were identified (Table S6). To gain a more extensive comprehension of the interplay between miRNAs and target genes, we chose 20 differentially expressed mRNAs (DEMs) associated with muscle development and 14 (DE)miRNAs that target them based on a thorough literature examination. The miRNA–mRNA interaction network was constructed using this information (Figure 4). It should be emphasized that some miRNAs, like miR-2478-z, miR-339-x, along with some newly discovered miRNAs (novel-m0036-3p and novel-m0037-3p), have the ability to target numerous genes associated with muscle development. The findings suggest that these miRNAs could have a significant impact on the regulation of muscle development.

### 3.5. Validation of Differentially Expressed miRNAs by qRT-PCR

**Figure 5:**
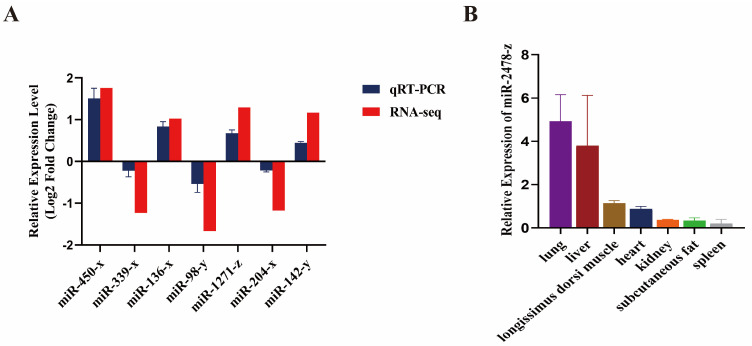
qRT−PCR detection of (DE)miRNAs. (**A**) qRT−PCR to verify the changes in miRNA expression between Gannan yaks and Jeryaks. (**B**) qRT−PCR analysis of miR−2478−z in heart, liver, spleen, lung, kidney, longissimus dorsi muscle and subcutaneous fat. Three biological replicates were used for each group. miRNAs with *U6* as an internal reference. The qRT−PCR data were determined using the 2^−ΔΔCt^ method. Validation data for miRNA sequencing results were further normalized to log2 (fold change). Data represent means ± standard error.

To establish the validity of the RNA-Seq outcomes, we evaluated seven differentially expressed miRNAs, including 4 up-regulated miRNAs (miR-450-x, miR-1271-z, miR-136-x, miR-142-y) and 3 down-regulated miRNAs (miR-339-x, miR-98-y, miR-204-x). The results showed that the miRNA expression patterns of Gannan yak and Jeryak were similar to the sequencing results (Figure 5A). This result further supports the repeatability and reliability of our sequencing data. And then we investigated the expression of miR-2478-z by qRT-PCR, which targets multiple genes involved in muscle growth. Our study found that miR-2478-z expression was relatively high in the longissimus dorsi muscle tissue (Figure 5B). Thus, these results also confirm the reliability of the reciprocal network and suggest that (DE)miRNAs play a regulatory role in muscle growth and development.

## 4. Discussion

Muscle growth is a multifaceted economic characteristic that not only impacts the quality of meat from livestock and poultry, but also plays a pivotal role in enhancing overall meat-production performance [35,36]. Compared to conventional bovines, yaks exhibit a slower growth rate and have a relatively modest production performance [37]. Therefore, the quest for strategies to optimize yak productivity continues to be a pressing inquiry in need of resolution. Jeryak, a hybrid breed resulting from crossbreeding of Gannan yak and Jersey cattle, have exhibited enhanced production capabilities in comparison to Gannan yak [38]. Hence, investigating the molecular regulatory mechanism underlying this phenomenon will contribute to enhancing the economic advantages associated with yaks.

miRNAs, crucial non-coding regulators in post-transcriptional regulation, exert their influence on a plethora of biological processes including cell proliferation, apoptosis, and the development of tumors [39]. A number of 230 miRNAs exhibited differential expression in the present study. Among these, a total of 135 (DE)miRNAs displayed noteworthy down-regulation in the longissimus dorsi muscle of Jeryak, whereas 95 (DE)miRNAs exhibited significant up-regulation. We performed GO and KEGG pathway enrichment analysis on differentially expressed miRNA target genes to investigate their potential functional roles. The GO enrichment results demonstrate that (DE)miRNAs primarily participate in the regulation of Wnt signaling and developmental processes. Additionally, KEGG pathway analysis revealed a significant enrichment of some (DE)miRNAs in key pathways including Ras signaling, MAPK signaling, Wnt signaling, and FoxO signaling. As a prominent pathway for miRNA target genes in Jeryak, the Ras signaling cascade has been documented to exert inhibitory effects on skeletal muscle myogenesis [40]. The MAPK signaling pathway has the capacity to regulate biological processes via multiple mechanisms of activating or inhibiting associated factors [41]. It has been found that activation of the p38/MAPK signaling pathway can contribute to a thickening of muscle fiber cross-sections and an increase in muscle length by regulating the protein content in adult muscle fibers after birth, thereby increasing total muscle mass [42,43]. A previous study has demonstrated that the Wnt signaling exerts direct control over the expression of myogenic regulatory factors (MRFs) during embryonic development in animals, thereby influencing muscle production [12]. Additionally, the FoXO signaling pathway is also involved in muscle growth and development, and it leads to skeletal muscle atrophy primarily through protein degradation [44]. Sandri et al. [45]. demonstrated that FoXO induces skeletal muscle atrophies by up-regulating the ubiquitin ligase Atrogin-1.

To further scrutinize the candidate miRNAs governing muscle growth and development in Jeryak, we successfully constructed a miRNA–mRNA interaction network based on the results of combined analysis of mRNAs and miRNAs. Significantly, the two newly identified miRNAs exhibited differential expressions in both species. The function of their target genes may mediate the effects of these miRNAs on muscle growth and development. Within the intricate network of interactions, novel-m0036-3p and novel-m0037-3p were both found to specifically target *SIX2*, *SIX1*, *NDRG4*, *STX4*, and *CTCF* genes. *SIX1* has been reported as a constituent of the vertebrate *SIX* gene family. During vertebrate skeletal myogenesis and development, the SIX1 transcription factor exerts a transcriptional regulatory effect on the myogenic determinant gene family, thereby indirectly modulating downstream muscle development-related genes through its regulation of individual members of MRFs [46]. Recent investigation reveals that excessive expression of the transcription factor SIX2 stimulates satellite cell growth in bovine skeletal muscles [47]. Zhu et al. [48]. reported that *NDRG2* treatment of C12C4 myoblasts activated the Akt/CREB signaling pathway and significantly up-regulated *MyoD* and *MyoG* gene expression, thereby promoting myoblast differentiation. *STX4*, also known as *syntaxin 4*, exhibits significant expression levels in skeletal muscle tissue. Yoo et al. [49] found that *STX4* enhanced the proliferation and differentiation of myoblasts by interacting with *CDO*. *CTCF* is critical for early embryonic development [50]. *CTCF* has been found to be a factor involved in the regulation of myogenesis, which promotes muscle differentiation mainly by coordinating with MRFs [51]. Interestingly, we also identified *SIX2*, *SIX1*, *NDRG4*, *STX4*, and *CTCF* as down-regulated DEMs. As a result, we speculated that these miRNAs might influence the growth and development of muscles by inhibiting the expression of DEMs associated with muscles.

In addition, it has been demonstrated in previous studies that a single miRNA can target multiple mRNA and, conversely, multiple miRNAs can target the same mRNAs [52]. Notably, our interaction network diagram revealed concurrent interactions of miR-339-x, miR-339-z, and miR-10926-z with *MICAL2*, as well as concurrent interactions of miR-421-y and miR-450-x with *MEF2A*. Therefore, we postulate that these miRNAs may exhibit specifically binding affinity towards target mRNAs, thereby exerting a discernible impact on the growth and development of Jeryak skeletal muscle. Subsequent investigations should encompass the validation of putative miRNA–mRNA targeting relationships to elucidate their underlying mechanisms governing skeletal muscle growth and development.

## 5. Conclusions

In this study, 230 (DE)miRNAs were identified by small RNA-Seq. The results of both GO and KEGG analyses have shown that target genes of (DE)miRNAs were principally engaged in signaling pathways related to muscle growth and development. Subsequently, 14 key miRNAs were screened by constructing miRNA–mRNA interaction networks, including miR-2478-z, miR-339-x, novel-m0036-3p, and novel-m0037-3p. These miRNAs may regulate muscle growth and development in Jeryaks by affecting their target genes.

## Figures and Tables

**Table 1:** Primers of miRNAs used for the RT-qPCR.

miRNAs	Forward (5′ → 3′)	Reverse (5′ → 3′)
miR-450-x	TTTTGCAATATGTTCCTGAAT	
miR-136-x	ACTCCATTTGTTTTGATGATGG	
miR-1271-z	CTTGGCACCTAGTAAGTACTCAA	
miR-142-y	TGTAGTGTTTCCTACTTTATGG	
miR-204-x	TTCCCTTTGTCATCCTATGCCT	
miR-98-y	CTATACAACTTACTACTTTCCT	
miR-339-x	TCCCTGTCCTCCAGGAGCTCACT	
U6	ACGGACAGGATTGACAGATT	TCGCTCCACCAACTAAGA

**Table 2:** Results of raw reads of Gannan Yaks (M1, M2 and M3) and Jeryaks (P1, P2 and P3) after quality control.

Samples	Clean Reads	High Quality	3′ Adapter Null	Insert Null	5′ Adapter Contaminants	PolyA (%)	Clean Tags
M1	17,408,914 (100%)	17,260,011 (99.1447%)	9612 (0.0557%)	108,519 (0.6287%)	31,981 (0.1853%)	321 (0.0019%)	16,255,077 (93.3721%)
M2	14,015,050 (100%)	13,899,769 (99.1774%)	6900 (0.0496%)	54,327 (0.3908%)	13,958 (0.1004%)	162 (0.0012%)	13,362,108 (95.3411%)
M3	16,597,591 (100%)	16,448,657 (99.1027%)	10,968 (0.0667%)	83,221 (0.5059%)	20,095 (0.1222%)	247 (0.0015%)	15,172,291 (91.4126%)
P1	14,318,748 (100%)	14,151,985 (98.8354%)	9475 (0.0670%)	59,453 (0.4201%)	7319 (0.0517%)	126 (0.0009%)	13,782,038 (96.2517%)
P2	9,195,586 (100%)	9,071,110 (98.6464%)	76,171 (0.8397%)	46,305 (0.5105%)	3837 (0.0423%)	76 (0.0008%)	8,756,687 (95.2271%)
P3	16,740,804 (100%)	16,610,388 (99.2210%)	66,607 (0.4010%)	74,568 (0.4489%)	6918 (0.0416%)	134 (0.0008%)	16,180,410 (96.6525%)

## Data Availability

The RNA-seq data from this study are available in GenBank Sequence Read Archive (SRA) database with accession number PRJNA1023680 and PRJNA1023693.

## Supplementary Materials

The following supporting information can be downloaded at: https://www.mdpi.com/article/10.3390/genes14122220/s1, Table S1: Distribution of small RNA types in Gannan yak and Jeryak; Table S2: Reference genome comparison rate; Table S3: Prediction of target genes of differentially expressed miRNAs; Table S4: GO enrichment analysis of differentially expressed miRNA target genes; Table S5: KEGG enrichment analysis of differentially expressed miRNA target genes; Table S6: Negative targeting relationship pairs of differentially expressed mRNAs and differentially expressed miRNAs.
